# Early nasal high-flow versus Venturi mask oxygen therapy after lung resection: a randomized trial

**DOI:** 10.1186/s13054-019-2361-5

**Published:** 2019-02-28

**Authors:** Mariano Alberto Pennisi, Giuseppe Bello, Maria Teresa Congedo, Luca Montini, Dania Nachira, Gian Maria Ferretti, Elisa Meacci, Elisabetta Gualtieri, Gennaro De Pascale, Domenico Luca Grieco, Stefano Margaritora, Massimo Antonelli

**Affiliations:** 1 Dipartimento delle Scienze dell’Emergenza, Anestesiologiche e della Rianimazione, UOC di Anestesia, Rianimazione, Terapia Intensiva e Tossicologia Clinica, Fondazione Policlinico Universitario A. Gemelli IRCCS, Rome, Italy; 2 Istituto di Anestesia e Rianimazione, Università Cattolica del Sacro Cuore, Rome, Italy; 3 Dipartimento delle Scienze Cardiovascolari e Toraciche, UOC di Chirurgia Toracica, Fondazione Policlinico Universitario A. Gemelli, IRCCS, Rome, Italy; 4 Istituto di Patologia Speciale Chirurgica, Università Cattolica del Sacro Cuore, Rome, Italy

**Keywords:** Hypoxemia, High-flow oxygen therapy, Noninvasive ventilation, Postoperative pulmonary complications, Thoracotomic lobectomy

## Abstract

**Background:**

Data on high-flow nasal oxygen after thoracic surgery are limited and confined to the comparison with low-flow oxygen. Different from low-flow oxygen, Venturi masks provide higher gas flow at a predetermined fraction of inspired oxygen (FiO_2_). We conducted a randomized trial to determine whether preemptive high-flow nasal oxygen reduces the incidence of postoperative hypoxemia after lung resection, as compared to Venturi mask oxygen therapy.

**Methods:**

In this single-center, randomized trial conducted in a teaching hospital in Italy, consecutive adult patients undergoing thoracotomic lung resection, who were not on long-term oxygen therapy, were randomly assigned to receive high-flow nasal or Venturi mask oxygen after extubation continuously for two postoperative days. The primary outcome was the incidence of postoperative hypoxemia (i.e., ratio of the partial pressure of arterial oxygen to FiO_2_ (PaO_2_/FiO_2_) lower than 300 mmHg) within four postoperative days.

**Results:**

Between September 2015 and April 2018, 96 patients were enrolled; 95 patients were analyzed (47 in high-flow group and 48 in Venturi mask group). In both groups, 38 patients (81% in the high-flow group and 79% in the Venturi mask group) developed postoperative hypoxemia, with an unadjusted odds ratio (OR) for the high-flow group of 1.11 [95% confidence interval (CI) 0.41–3] (*p* = 0.84). No inter-group differences were found in the degree of dyspnea nor in the proportion of patients needing oxygen therapy after treatment discontinuation (OR 1.34 [95% CI 0.60–3]), experiencing pulmonary complications (OR 1.29 [95% CI 0.51–3.25]) or requiring ventilatory support (OR 0.67 [95% CI 0.11–4.18]). Post hoc analyses revealed that PaO_2_/FiO_2_ during the study was not different between groups (*p* = 0.92), but patients receiving high-flow nasal oxygen had lower arterial pressure of carbon dioxide, with a mean inter-group difference of 2 mmHg [95% CI 0.5–3.4] (*p* = 0.009), and were burdened by a lower risk of postoperative hypercapnia (adjusted OR 0.18 [95% CI 0.06–0.54], *p* = 0.002).

**Conclusions:**

When compared to Venturi mask after thoracotomic lung resection, preemptive high-flow nasal oxygen did not reduce the incidence of postoperative hypoxemia nor improved other analyzed outcomes. Further adequately powered investigations in this setting are warranted to establish whether high-flow nasal oxygen may yield clinical benefit on carbon dioxide clearance.

**Trial registration:**

ClinicalTrials.gov, NCT02544477. Registered 9 September 2015.

**Electronic supplementary material:**

The online version of this article (10.1186/s13054-019-2361-5) contains supplementary material, which is available to authorized users.

## Background

Patients undergoing lung resection are jeopardized by relevant postoperative morbidity and mortality [1–3]. Acute respiratory failure is the most common life-threatening complication after thoracic surgery. Supplemental oxygen is often needed to improve arterial oxygenation in the postoperative period: despite it is effective in treating most cases of hypoxemia, patients with low ventilation-perfusion ratio may be only partially responsive to an increase in oxygen concentration. Noninvasive ventilation (NIV) has been proposed to prevent/treat respiratory failure after lung resection [4–8], but its routine use in clinical practice requires personnel expertise and technological resources that may not be available in all post-anesthesia care units and surgical wards. In addition, in the early postoperative period, delivery of positive pressure in the airways and eventual patient-ventilator asynchronies during assisted ventilation may pose a risk to the tightness of bronchial anastomosis by means of uncontrolled swings in the transmural pressure of the airways.

The use of nasal cannula to deliver high flow rates of heated and humidified gas at a predetermined fraction of inspired oxygen (FiO_2_) is an attractive alternative to conventional oxygen therapy and, possibly, to NIV [9]. The beneficial effects of high-flow nasal cannula (HFNC) include (a) delivery of high flows, that better match patients’ peak inspiratory flow, finally enabling administration of set FiO_2_; (b) provision of a small degree of positive pressure in the airways, that increases end-expiratory lung volume; (c) washout of nasopharyngeal dead space, which enhances carbon dioxide (CO_2_) removal; and (e) good tolerance and comfort [10–14]. HFNC, as compared to low-flow oxygen, prevents respiratory failure after extubation in the intensive care unit and is as effective as NIV after cardiothoracic surgery and in patients with difficult separation from mechanical ventilation [15–19]. Also when compared to oxygen therapy with Venturi mask, which itself produces mid-to-high flows of gas at predetermined FiO_2_ due to an air entrainment mechanism, HFNC improves oxygenation, comfort, and CO_2_ clearance, possibly facilitating weaning from mechanical ventilation in critically ill patients [20].

No study ever compared HFNC and Venturi mask for oxygen therapy after thoracic surgery: we hereby report the results of a randomized trial conducted to determine whether early treatment with HFNC, as compared to Venturi mask, can prevent the development of clinically relevant hypoxemia after thoracotomic lung lobectomy.

## Methods

### Study design

This single-center, open-label, randomized controlled study was conducted in the post-anesthesia care unit, surgical intensive care unit, and thoracic surgical ward of a tertiary university hospital in Italy, between September 2015 and April 2018. The protocol was approved by the local ethics committee and was registered on ClinicalTrials.gov (NCT02544477) before trial initiation. The study was conducted in accordance with the declaration of Helsinki, and written informed consent was obtained from all enrolled subjects according to committee recommendations.

### Participants

All adult patients scheduled for elective thoracotomic pulmonary lobar resection for malignant disease were eligible for study inclusion. Exclusion criteria were refusal of informed consent, pregnancy, body mass index ≥ 35 kg/m^2^, history of obstructive sleep apnea syndrome, long-term oxygen therapy due to chronic pulmonary disease, presence of tracheostomy, and any nasal/facial defect that could impede HFNC or Venturi mask use.

Patients enrolled in the study were randomized to receive oxygen therapy by a Venturi face mask or a treatment with HFNC continuously over the course of 48 h after surgery. A computer-generated random allocation list was used to allocate enrolled patients to study arms.

### Patient management during surgery

All enrolled patients received general anesthesia according to the following standard protocols: induction with propofol 2–3 mg/kg, fentanyl 1.5–2.5 mcg/kg, and rocuronium bromide 0.9 mg/kg; maintenance provided by sevoflurane titrated to keep bi-spectral index values between 40 and 60%, continuous infusion remifentanil 0.05–0.4 mcg/kg/min, repeated boluses of rocuronium bromide 0.02 mg kg^−1^ to maintain a train of four of 1–3 by neuromuscular monitoring, and 3–5 ml/kg/h of intravenous crystalloids and antibiotic prophylaxis; and postoperative analgesia was obtained by intercostal nerve block at the end of the procedure and paracetamol at a standard dose for the first three postoperative days. During surgery, all patients were ventilated with a tidal volume of 6–8 ml/kg of predicted body weight [21] for two-lung ventilation and of 5 ml/kg for one-lung ventilation; PEEP was set at 5 cmH_2_O throughout the whole surgical procedure. Recruitment maneuvers were performed once (i.e., after lobectomy) in all patients.

At the end of surgery, patients were extubated as the following criteria were met: spontaneous respiratory activity with exhaled tidal volume between 5 and 8 ml/kg; respiratory frequency ranging between 12 and 30 breaths/min; absence of residual neuromuscular blockade, as assessed by train-of-four monitoring; peripheral oxygen saturation (SpO_2_) ≥ 92%; hemodynamic stability (heart rate < 120/min; systolic blood pressure between 90 and 160 mmHg; no signs of cardiac ischemia, no hemodynamically significant arrhythmias and absence of catecholamines); body temperature ≥ 36 °C; adequate cough reflex; and absence of copious secretions.

After extubation, enrolled patients were transferred to either the post-anesthesia care unit or the intensive care unit, according to the decision of the attending anesthesiologist, who was not aware of the randomization arm. According to department guidelines, early intensive care unit admission was reserved to American Society of Anesthesiologists’ three patients who were deemed at high-risk of postoperative complications. Patients treated in the post-anesthesia care unit were transferred to the surgical ward within 4–8 h after surgery, unless deemed clinically inappropriate. Patients treated in the intensive care unit were transferred to the surgical ward on postoperative day 1, unless clinically contraindicated.

### Study treatments

All patients had to undergo the assigned treatment within 30 min after extubation.

Patients in the control group received oxygen therapy via a Venturi mask (OS/60 K, FIAB, Florence, Italy); pure O_2_ flow was set depending on the needed FiO_2_ according to manufacturer recommendations. Patients in the intervention group received HFNC by AIRVO™ (Fisher & Paykel Healthcare Ltd., Auckland, New Zealand). The initial flow rate was 50 l/min and was eventually diminished in case of intolerance. Humidification chamber temperature was set at 37 °C and eventually diminished in case of intolerance.

In both groups, SpO_2_ was monitored continuously and FiO_2_ was titrated on an hour basis to maintain SpO_2_ between 92% and 98%. The assigned treatment was administered continuously until day 2 after surgery, 9.00 a.m., when patients were assessed for treatment interruption; study treatments were discontinued and patients were deemed weaned from oxygen therapy as the following criteria were met: respiratory rate ≤ 35 breaths/min; no recruitment of accessory muscles during calm breathing; hemodynamic stability (heart rate < 120/min; systolic blood pressure between 90 and 160 mmHg; no signs of cardiac ischemia, no hemodynamically significant arrhythmias, and absence of catecholamines); and core body temperature < 38.5 °C. After day 2, in case of failure to be weaned from oxygen therapy, all enrolled patients received Venturi Mask oxygen therapy, as long as deemed appropriate by the attending physician.

In the surgical ward, patients from both groups underwent a standard physiotherapy protocol: over the initial 24 h, this consisted of upright positioning, sitting on the edge of the bed or on the chair, non-resistance leg exercises, and lung expansion maneuvers twice a day (i.e., deep diaphragmatic breathing, thoracic expansion exercises, and incentive spirometry). As soon as the patient was weaned from oxygen therapy, a walking program was also adopted.

### Measurements

Baseline blood gas analysis was obtained in the preoperative period. Postoperative blood gas analyses and dyspnea assessment were performed 1, 3, and 24 h after extubation and then on a daily basis up to day 4. Chest X-ray was obtained 2 h after surgery and then on a daily basis up to postoperative day 3.

Self-assessment of dyspnea (i.e., respiratory discomfort-shortness of breath) was performed by a visual analog scale (VAS) ranging from 0 (no dyspnea) to 10 (maximum dyspnea) (in Additional file 1: Figure S1).

### Endpoints

The primary endpoint of the study was the overall incidence of patients developing clinically relevant hypoxemia (i.e., ratio of the partial pressure of arterial oxygen (PaO_2_) to FiO_2_ (PaO_2_/FiO_2_) < 300 mmHg) during the first four postoperative days.

Secondary outcomes were (i) the need for supplemental oxygen after study treatment discontinuation and within 7 days from randomization (i.e., a peripheral arterial oxygen saturation (SpO_2_) < 93% while breathing on room air); (ii) the occurrence of postoperative severe acute respiratory failure requiring ventilatory support; (iii) the degree of dyspnea over the course of the first four postoperative days; and (iv) the rate of pulmonary complications within 7 days after surgery.

In non-prespecified post hoc analyses, we also assessed (i) the overall incidence of patients developing moderate-to-severe hypoxemia (PaO_2_/FiO_2_ < 200 mmHg) over the first 96 h after surgery; (ii) the cumulative incidence of clinically relevant hypercapnia (i.e., PaCO_2_ > 45 mmHg) over the course of the first four postoperative days; (iii) PaO_2_/FiO_2_ and PaCO_2_ over the course of the first four postoperative days; (iv) the length of hospital stay; and (v) all-cause 30-day mortality.

Postoperative severe acute respiratory failure requiring ventilatory support was defined as the presence of at least two of the followings: respiratory acidosis (arterial pH ≤ 7.35 with PaCO_2_ > 45 mmHg); SpO_2_ < 90% or PaO_2_ < 60 mmHg at an FiO_2_ ≥ 0.5; respiratory frequency > 35/min; altered state of consciousness; and clinical signs of respiratory muscle fatigue [22]. Respiratory failure was initially treated with NIV, except when endotracheal intubation was required (i.e., cardiac arrest, loss of consciousness, psychomotor agitation, massive aspiration, persistent inability to remove respiratory secretions, heart rate < 50/min with loss of alertness, and severe hemodynamic instability without response to fluids and vasoactive drugs [22]). Patients with worsening blood gases and/or persistent tachypnea (respiratory rate > 35 breaths/min) despite NIV received endotracheal intubation.

Postoperative pulmonary complications were defined as sub-lobar or lobar atelectasis, detected by the chest X-rays and scored using the radiological atelectasis score equal or greater than two [23]; nosocomial pneumonia (new-onset or progressive pulmonary infiltrates with at least two of the following: purulent respiratory secretions, temperature > 38 °C or < 36 °C, and white blood cell count > 12,000/mm^3^ or < 4000/mm^3^) [24]. Non-pulmonary complications included new-onset cardiac arrhythmias, cardiac ischemia, hemodynamic instability requiring fluid or vasoactive resuscitation, hyperlactatemia, and metabolic acidosis.

The post hoc analyses on PaCO_2_ and hypercapnia development were conducted under the light of the most recent evidence suggesting a relevant effect of HFNC on CO_2_ washout in the upper airways [10–12, 25–27]. Results on these endpoints should be considered merely exploratory in nature.

### Statistical analysis

Data on the rate of patients experiencing postoperative hypoxemia (defined as a PaO_2_/FiO_2_ ratio < 300 mmHg within 96 h after surgery) during Venturi mask oxygen therapy after lung resection were lacking at the time of study design, but it was known that 50% of them show a PaO_2_/FiO_2_ < 320 mmHg 24 h after surgery while on low-flow oxygen [28]. Using a conservative approach, we hypothesized a 45%-incidence of postoperative hypoxemia in the Venturi mask group, and we estimated that 45 patients per group were needed to detect a 60% relative reduction in the rate of the primary endpoint in the intervention group (estimated absolute risk in the intervention group, 18%), with a type I error set at 5% and statistical power of 80%. Given an attrition rate lower than 5%, mostly due to protocol violations and crossover between treatments, we planned to enroll 94 patients.

The analysis was conducted on a “modified intention-to-treat” population that included all patients who underwent the allocated treatment for at least 6 h.

Distribution normality was assessed with the Kolmogorov-Smirnov test. Continuous variables with normal distribution are reported as means (± standard deviation), whilst those with non-normal distributions were expressed as medians (interquartile ranges).

Analysis on the primary efficacy criterion and for other categorical outcomes was performed with the χ^2^ test, or Fisher’s exact test, as appropriate: Cochran–Mantel–Haenszel statistics are reported for all these results. For other relevant outcomes whose distribution was statistically different in the two groups at the univariate analysis, a logistic regression model was conducted: all variables with *p* ≤ 0.20 at the univariate analysis were included. Kaplan–Meier curves were plotted to assess the time from enrollment to the primary endpoint or relevant secondary outcomes by means of the log-rank test; for secondary outcomes, Cox regression analysis was also conducted to confirm the independent effect of the treatment on the time from enrollment to occurrence of the endpoint: all variables with a log-rank *p* ≤ 0.20 were included in the model. Two-way analysis of variance (ANOVA) for repeated measures with Bonferroni correction was used to determine the differences in PaO_2_/FiO_2_ ratio, PaCO_2_, and dyspnea in the two groups. Comparisons between groups regarding these variables at each study timepoint were performed with the Student’s *t* test or Mann-Whitney test, as appropriate. Mean difference and 95% confidence interval (95% CI) are reported for most significant results.

Two-tail *p* values ≤ 0.05 were considered significant. Statistical analysis was performed with SPSS software package (SPSS Inc. Released 2009. PASW Statistics for Windows, Version 18.0. Chicago: SPSS Inc.).

## Results

Between September 2015 and April 2018, of the 522 patients undergoing thoracic surgery for lung cancer, 99 patients were eligible for inclusion in the study and 96 underwent randomization. All enrolled patients were successfully extubated at the end of surgery and received the allocated treatment within 30 min after extubation.

**Fig. 1:**
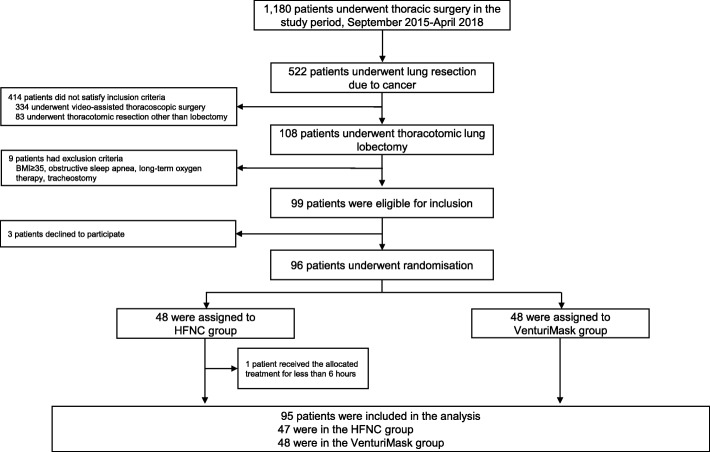
Consort flow diagram

One patient from the HFNC group was not included in the “modified intention-to-treat” population because, due to intolerance to the device, the patient received the allocated treatment for less than 6 h. Data from 95 patients (47 in the HFNC group and 48 in the Venturi mask group) were analyzed (Fig. 1).

Demographics, most relevant clinical characteristics, main comorbidities, preoperative arterial blood gases and respiratory function, and surgical procedures are reported in Table 1 and were well balanced in the two study groups. No patient had preoperative PaCO_2_ exceeding 45 mmHg. All patients had undergone thoracotomic lung lobar resection; mean duration of mechanical ventilation before extubation was 215 min ± 59 in the HFNC group and 222 ± 119 in the Venturi mask group.

**Table 1:** Demographics and baseline clinical characteristics of enrolled patients

Characteristic		HFNC (*n* = 47)	Venturi mask (*n* = 48)
Age, years		66 ± 10	68 ± 9
Female sex	*n* (%)	20 (43)	21 (48)
Height, cm		165 ± 9	166 ± 9
Weight, kg		72 ± 14	74 ± 15
Body mass index, kg/m^2^		26 ± 4	27 ± 4
American Society of Anesthesiologist physical status	*n* (%)		
I		1 (2)	2 (4)
II		33 (70)	31 (75)
III		13 (28)	15 (21)
Comorbidities	*n* (%)		
Induction therapy		6 (13)	6 (13)
History of cardiac failure		4 (9)	7 (15)
History of ischemic heart disease		5 (11)	11 (23)
Chronic obstructive pulmonary disease¶		25 (53)	32 (67)
Pulmonary infections in the preceding month		12 (26)	11 (23)
Active smoking		23 (49)	28 (58)
Diabetes		4 (9)	7 (15)
Preoperative respiratory function			
FEV_1_, liters		2.2 ± 0.7	2.2 ± 0.7
Forced vital capacity, liters		3.2 ± 0.9	3.3 ± 0.8
Tiffenau index, %		72 ± 11	70 ± 10
Preoperative arterial blood gases			
PaO_2_, mmHg		85 ± 10	83 ± 10
PaCO_2_, mmHg		37 ± 3	37 ± 3
Patients with PaCO_2_ ≥ 45 mmHg	*n* (%)	0	0
Side of surgery	*n* (%)7		
Right		30 (64)	29 (61)
Left		17 (36)	19 (39)
Site of surgery	*n* (%)		
Upper lobe		24 (51)	28 (59)
Middle lobe		3 (6)	5 (10)
Lower lobe		20 (42)	15 (31)
Intraoperative tidal volume			
During two-lung ventilation			
ml		375 (350–450)	425 (369–456)
ml/kg of predicted body weight		6.7 (6.4–7)	6.9 (6.5–7.3)
During one-lung ventilation			
ml		300 (263–325)	313 (250–350)
ml/kg of predicted body weight		5 (4.9–5.2)	5 (4.9–5.2)
Duration of mechanical ventilation, minutes		215 ± 59	222 ± 119
Duration of surgery, minutes		175. ± 66	172 ± 56
Intraoperative blood loss, ml		50 (0–200)	90 (0–163)
Intraoperative crystalloids, ml		900 (500–1500)	650 (500–1000)
Post-surgical management*	*n* (%)		
Post-anesthesia care unit		42 (89)	39 (81)
Length of PACU stay, hours		6 (5–7)	6 (5–7)
Intensive care unit		5 (11)	9 (19)
Length of ICU stay, days		1 (1–3)	1 (1–3)

Data are displayed as mean ± standard deviation or median (interquartile range), if not otherwise specified

*HFNC* high-flow nasal cannula, *PaCO*_*2*_ arterial partial pressure of carbon dioxide, *PaO*_*2*_ arterial partial pressure of oxygen

¶Defined on the basis of the GOLD definitions [41]

*The decision on whether the patient had to be transferred to the post-anesthesia or intensive care unit after extubation was taken by the attending anesthesiologist, who was aware of patient’s inclusion in the trial but not of the randomization arm

Mean HFNC FiO_2_ at treatment start was 41% ± 5, with gas flow set at 50 l/min in all patients. In the Venturi mask group, mean FiO_2_ at treatment initiation was 39% ± 4 with a pure oxygen flow of 8 ± 1 l/min, which corresponded to a delivered nominal gas flow of 33 ± 2 l/min. Mean (CI 95%) nominal FiO_2_ during the assigned treatment was 39% [29–31] in the HFNC group and 38% [29, 30, 32] in the Venturi mask group (*p* = 0.23, in Additional file 1: Figure S2).

Main results of the study are reported in Table 2.

**Table 2:** Primary and secondary outcomes, according to the study group

Outcome	Study group		*p* value	Odds ratio or mean difference (95% CI)
	HFNC (*n* = 47)	Venturi mask (*n* = 48)		
Primary outcome				
Incidence of postoperative hypoxemia (PaO_2_/FiO_2_ < 300 mmHg)				
Unadjusted analysis			0.84	1.11 (0.41–3)
No. of patients	38	38		
% of patients (95% CI)	81 (69–93)	79 (67–91)		
Secondary outcomes				
Need for supplemental oxygen after treatment discontinuation				
Unadjusted analysis			0.48	1.34 (0.60–3)
No. of patients	24	21		
% of patients (95% CI)	51 (36–66)	44 (29–58)		
Incidence of postoperative respiratory failure requiring ventilatory support*				
Unadjusted analysis			> 0.999	0.67 (0.11–4.18)
No. of patients	2	3		
% of patients (95% CI)	4 (0–11)	6 (0–13)		
Incidence of postoperative pulmonary complications				
Unadjusted analysis			0.64	1.29 (0.51–3.25)
No. of patients	13	11		
% of patients (95% CI)	28 (14–41)	23 (11–35)		
Mean dyspnea during the first 4 postoperative days				
ANOVA for repeated measures			0.97	0 (− 1–1)
Mean	2.2	2.3		
95% CI	1.5–2.9	1.5–3		
Other secondary outcomes				
Incidence of moderate-to-severe postoperative hypoxemia (PaO_2_/FiO_2_ < 200 mmHg)				
Unadjusted analysis			0.67	1.24 (0.54–2.88)
No. of patients	18	16		
% of patients (95% CI)	38 (24–53)	33 (20–47)		
Incidence of postoperative hypercapnia (PaCO_2_ > 45 mmHg)				
Unadjusted analysis			0.004	0.24 (0.09–0.63)
No. of patients	8	22		
% of patients (95% CI)	17 (6–28)	46 (31–60)		
Adjusted analysis¶			0.002	0.18 (0.06–0.54)
Mean PaO_2_/FiO_2_				
In the first four postoperative days				
ANOVA for repeated measures			0.92	1 (− 30–33)
Mean	300	299		
95% CI	279–322	276–322		
During assigned treatments (two postoperative days)				
ANOVA for repeated measures			0.72	5 (− 24–35)
Mean	301	296		
95% CI	281–321	274–317		
Mean PaCO_2_				
In the first four postoperative days				
ANOVA for repeated measures			0.015	− 1.7 (− 3 to − 0.3)
Mean	38.9	40.6		
95% CI	38–39.8	39.6–41.5		
During assigned treatments (two postoperative days)				
ANOVA for repeated measures				
Mean	39.7	41.6	0.009	− 2 (− 3.4 to − 0.5)
95% CI	38.7–40.6	40.5–42.7		
Incidence of overall postoperative complications				
Unadjusted analysis			0.61	1.25 (0.53–2.98)
No. of patients	16	14		
% of patients (95% CI)	34 (20–48)	29 (16–43)		
Length of hospital stay, days				
Unadjusted analysis			0.83	− 2 (− 8–4)
Median	6	6		
Interquartile range	5–7	5–7		
28-day mortality				
Unadjusted analysis			n/a	
No. of patients	0	0		

¶The analysis was adjusted for age, history of clinically documented pulmonary infections in the month preceding surgery and preoperative PaCO_2_

*Four patients needed NIV (three patients in the Venturi mask group and one in the HFNC group) and two patients underwent endotracheal intubation (one in each group)

**Fig. 2:**
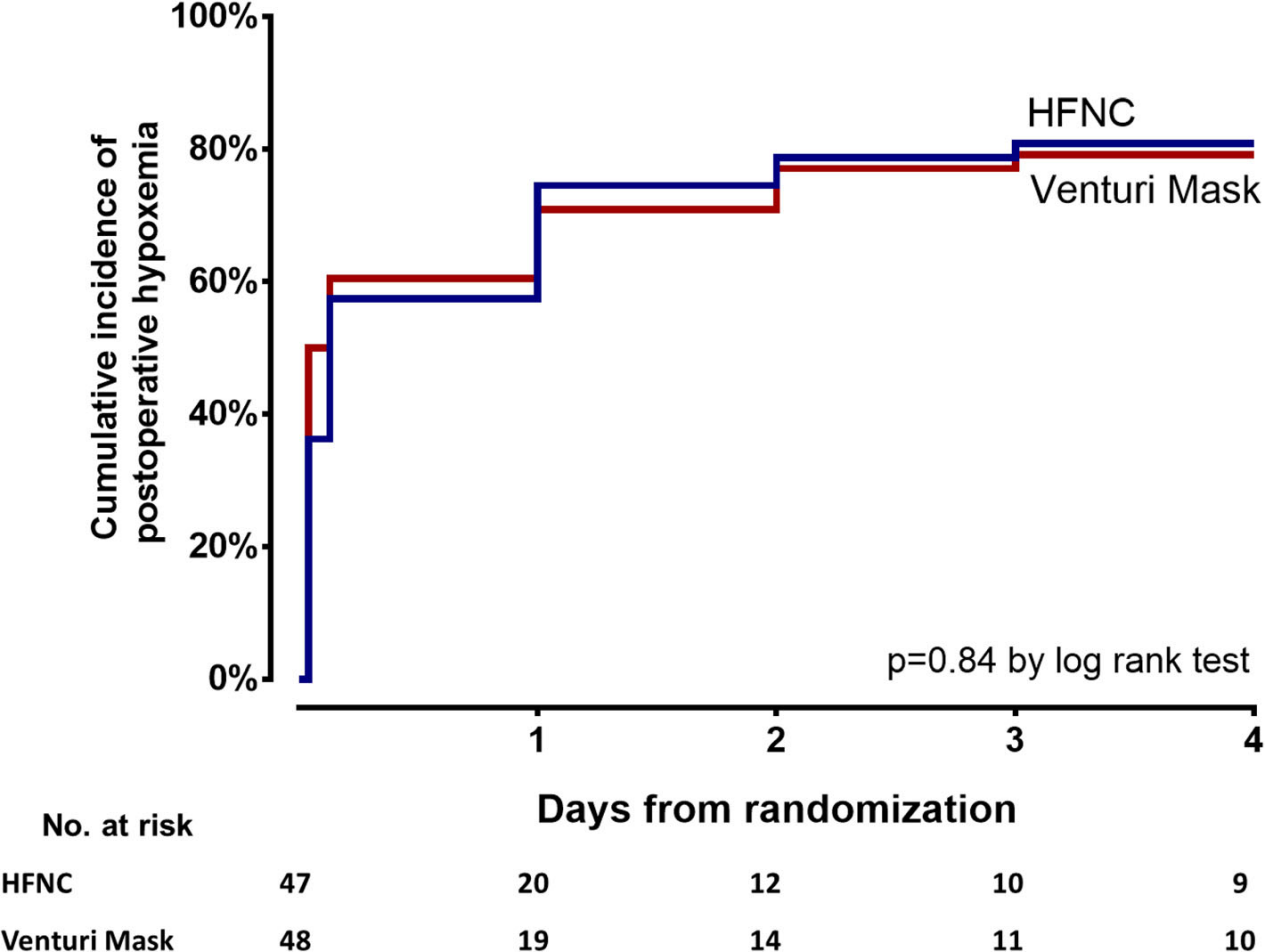
Kaplan–Meier plots of the cumulative incidence of postoperative hypoxemia

Both in the HFNC and Venturi mask groups, 38 patients developed hypoxemia within 96 h after extubation (81% vs. 79%, unadjusted odds ratio (OR) [95% CI] for HFNC 1.11 [0.41–3, *p* = 0.84) (Fig. 2). Similarly, the incidence of moderate-to-severe postoperative hypoxemia was not different between groups: 38% the in HFNC group vs. 33% in the Venturi mask group (unadjusted OR 1.24 [0.54–2.88], *p* = 0.67) (in Additional file 1: Figure S3).

No inter-group differences were found regarding the main secondary outcomes that were analyzed (i.e., the incidence of acute respiratory failure requiring ventilatory support, pulmonary complications, number of patients requiring oxygen therapy after treatment discontinuation, and the dyspnea during the study (in Additional file 1: Figure S4)).

**Fig. 3:**
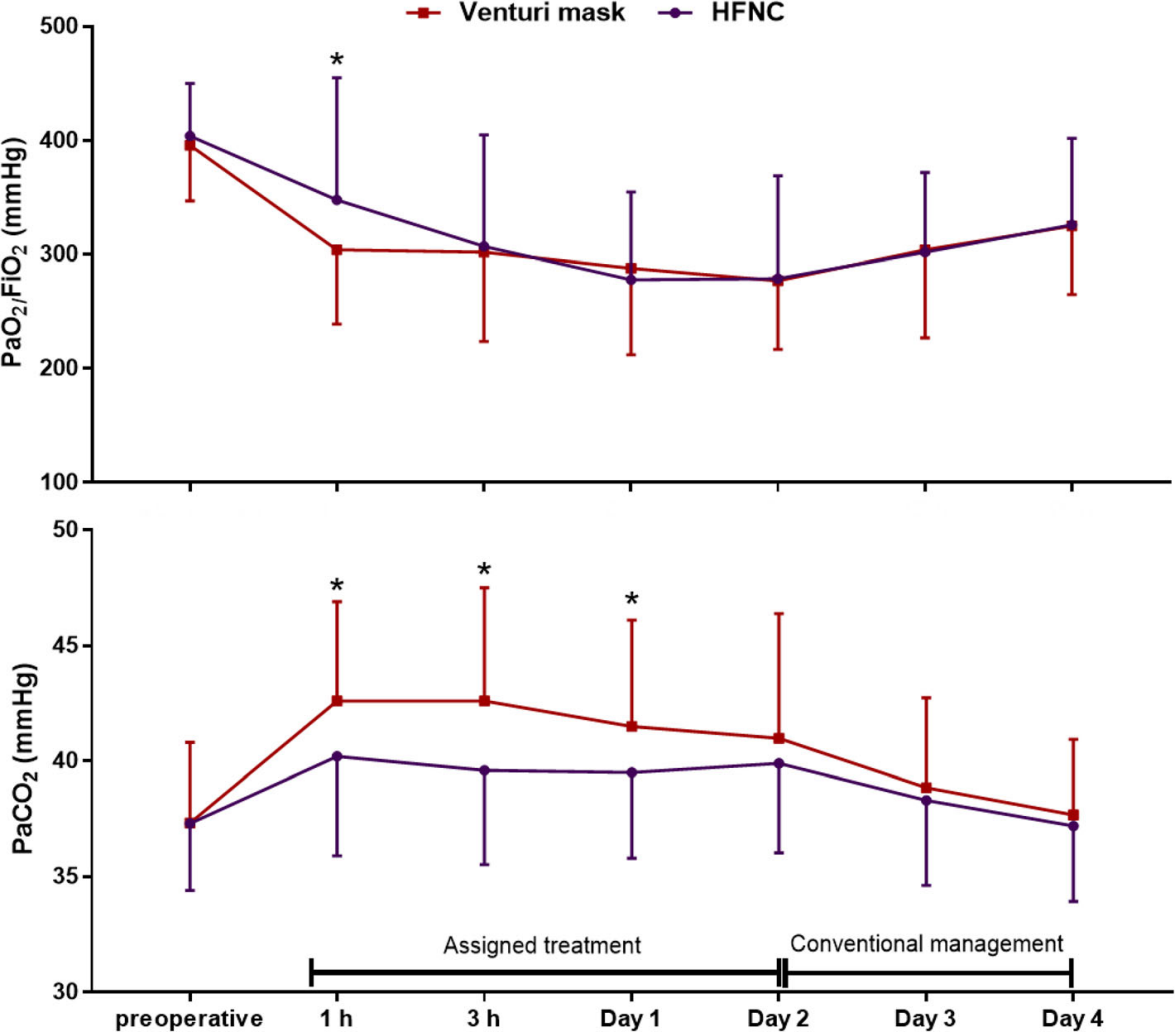
Postoperative PaO_2_/FiO_2_ ratio and PaCO_2_ in the two study groups. Results are expressed as means and standard deviation. No differences were detected in the PaO_2_/FiO_2_ ratio (ANOVA *p* = 0.92). Patients in the HFNC group showed lower PaCO_2_ over the entire course of the study (ANOVA *p* = 0.015), with a mean difference between study treatments of 1.7 mmHg [95% CI 0.3–3]. This difference was particularly evident while the assigned treatments were administered, with a mean difference between groups of 2 mmHg [95% CI 0.5–3.4] (ANOVA *p* = 0.009). *Indicates *p* < 0.05 for the comparison between HFNC and Venturi mask at the single timepoint

Over the course of the first 96 h after surgery, PaO_2_/FiO_2_ was not different between groups (*p* = 0.92), although patients in the HFNC showed higher PaO_2_/FiO_2_ 1 h after surgery: 347 vs. 304 mmHg, mean difference of 44 mmHg [95% CI 8–80] (*p* = 0.017) (Fig. 3).

**Fig. 4:**
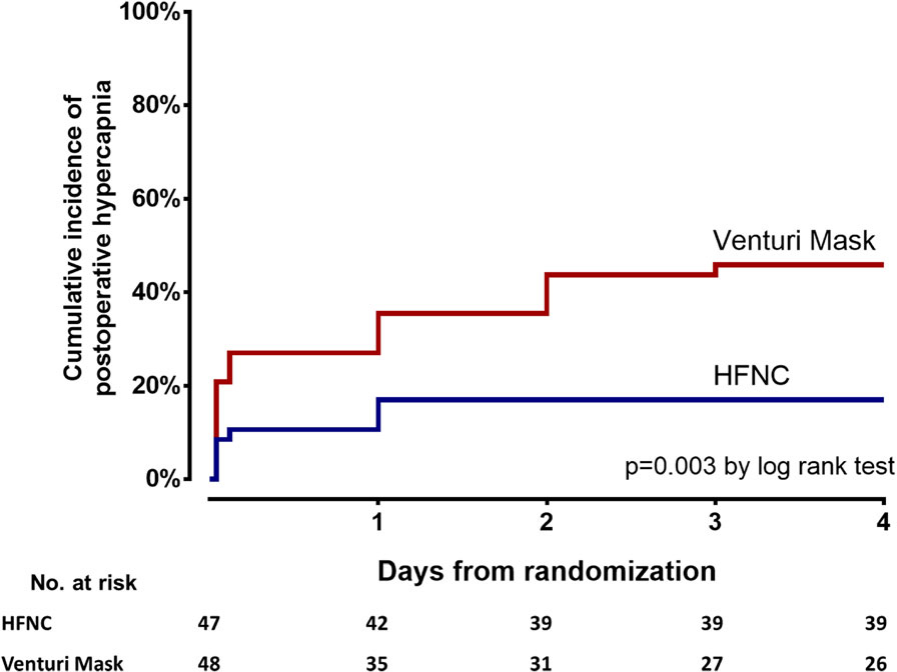
Kaplan–Meier plots of the cumulative incidence of postoperative hypercapnia in the two study groups. The inter-group difference remained significant after adjustment for age, history of clinically documented pulmonary infections in the month preceding surgery, and preoperative PaCO_2_, with a hazard ratio for HFNC of 0.33 [0.14–0.74] (*p* = 0.007). Please note that this analysis was not prespecified and should be considered exploratory in nature.

During the study, patients undergoing HFNC showed lower PaCO_2_, with a mean difference between groups of 2 mmHg [95% CI 0.5–3.4] (Fig. 3). Eight patients (17%) in the HFNC group and 22 (46%) in the Venturi mask group developed hypercapnia (PaCO_2_ > 45 mmHg) within 96 h after randomization (*p* = 0.004): this finding remained significant after adjustment for possible confounders, with an OR of 0.18 [0.06–0.54], *p* = 0.002 (univariate analysis reported in Additional file 2 Kaplan–Meier showed in Fig. 4). Importantly, PaO_2_ and SpO_2_ did not differ between groups in any of the analyzed study steps (*p* = 0.49 and *p* = 0.80, respectively).

## Discussion

In this randomized trial conducted in patients undergoing thoracotomic lung lobectomy, preemptive use of HFNC after extubation, as compared to Venturi mask oxygen therapy, did not result in lower incidence of postoperative hypoxemia nor had any effect on other prespecified secondary outcomes (incidence of postoperative respiratory complications and respiratory failure requiring ventilatory support, persistent need for oxygen therapy on postoperative day 2, postoperative dyspnea).

Because of anesthesia-induced pulmonary atelectasis, the development of surgical pneumothorax and the reduction in functional residual capacity generated by lung resection, hypoxemia represents a life-threatening complication and the leading cause of death in patients after thoracic surgery [28, 33].

Previous investigations in surgical and critically ill patients showed that HFNC may be of benefit after extubation. Favorable results on the preemptive use of HFNC, as compared to low-flow oxygen, have been reported in critically ill patients [16, 17], while the evidence appears conflicting in the postoperative period. After cardiac surgery, the use of HFNC does not yield improvement in oxygenation nor reduces the rate of atelectasis, but may be associated to a lower need for respiratory support escalation [23, 34] and may perform as well as noninvasive ventilation among patients at high risk for acute respiratory failure [19, 35]. After abdominal surgery, HFNC therapy does not reduce the incidence of hypoxemia or pulmonary complications in the postoperative period [36].

Differently, encouraging results emerge from trials comparing HFNC and low-flow oxygen in patients after thoracic surgery, who represent a specific population deemed at high risk for respiratory complications. When tested after thoracoscopic lung resection, HFNC was shown to improve oxygenation and decrease the rate of postoperative complications [37], with a possibly reduced postoperative length of stay [38].

If different studies addressed the differences between post-extubation HFNC and low-flow oxygen [39, 40], data on the clinical comparison between HFNC and Venturi mask are limited and confined to the setting of critical illness [20]. Thanks to the air entrainment effect, Venturi masks are capable to provide the patient with a nominal gas flow often above 30 l/min, at predetermined FiO_2_. This allows delivery of mid-to-high gas flow with essentially stable FiO_2_ and makes the Venturi system an optimal, easy-to-use, “conventional strategy” for oxygen therapy in patients with high respiratory demand [32]. Accordingly, while the benefit on oxygenation by HFNC over low-flow oxygen is immediate and pronounced [12], it appears milder and delayed over Venturi mask [20]. This aspect contributes to explain why, despite improving weaning outcome in critical patients if compared to low-flow oxygen [17] (and performing as well as NIV in high-risk patients [16]), the clinical effect of HFNC might be limited when compared to Venturi mask, as shown in the present investigation. Importantly, whether any difference exists between HFNC and Venturi masks in terms of weaning outcome in the intensive care unit will be clarified by the results of a recent large randomized study (ClinicalTrials.gov NCT02107183).

The lack of a significant effect of HFNC on oxygenation and on the development of postoperative hypoxemia is not conflicting with what reported by Maggiore et al., who showed that HFNC could provide an improvement in oxygenation only after 24 h of treatment [20]. In that study, that was conducted in the critical care setting, patients were studied in the weaning from mechanical ventilation after acute respiratory failure and were hypoxemic at study entry, while our patients quickly recovered after surgery, possibly mitigating any delayed effect on oxygenation resulting from the technique used to deliver oxygen.

In our study, PaCO_2_ was lower in patients undergoing HFNC than in those receiving Venturi mask. Consistently, although this endpoint was exploratory in nature, we report a lower rate of postoperative hypercapnia in patients treated with HFNC. The benefit by HFNC on CO_2_ is a well-known effect of the treatment and results from washout of the upper airways and lower CO_2_ production by respiratory muscles [11, 12, 20, 29]. Moreover, more than half of our study population had chronic obstructive pulmonary disease (COPD): patients with chronic respiratory failure are prone to develop respiratory complications related to CO_2_ retention and benefit from HFNC also when in stable clinical conditions [25–27].

This study has limitations. First, it was conducted in a single center, and the generalizability of our results may be limited; however, patients were enrolled according to well-defined inclusion criteria, which should enhance the reproducibility of our findings. Second, it was not possible to blind staff and participants to treatment allocation: nonetheless, we took into account clinically objectifiable endpoints that were unlikely affected by assessors’ unblinding. Third, we did not measure effectively delivered FiO_2_, as performed elsewhere [30]. As a result, the calculation of PaO_2_/FiO_2_ ratios could have been subject to errors [31]. Nevertheless, our approach is clinically reproducible, and the final mid-to-high flows produced by air entrainment during Venturi mask could have helped obtain parameters comparable with those obtained with HFNC. Fourth, we do not provide results about the respiratory rate, work-of-breathing or inspiratory effort, which were however not easy to obtain in a reliable fashion in the setting of a clinical trial. Finally, the analysis on the development of postoperative hypercapnia was conducted under the light of recent evidence indicating a clinical effect of HFNC on CO_2_ washout from upper airways [10–12, 25–27] and was not prespecified: these results, although confirmed after adjustment for possible confounders, should be seen as hypothesis-generating rather than conclusive.

## Conclusions

As compared with Venturi mask oxygen therapy in patients who undergo thoracotomic pulmonary lobectomy, preemptive HFNC early after extubation does not reduce the incidence of postoperative hypoxemia, does not limit the occurrence of postoperative pulmonary complications, and does not relieve dyspnea. Because recent data suggest that a major effect of HFNC is mediated by upper airways’ washout, a possible benefit by preemptive HFNC on CO_2_ clearance may be of interest among patients undergoing thoracic surgery and warrants further adequately powered investigations.

## Additional files

Additional file 1: **Figure S1.** Visual analog scale for the assessment of patient’s dyspnea. **Figure S2.** Mean (standard deviation) FiO_2_ in the two study groups (*p* = 0.23 for the inter-group comparison). **Figure S3.** Kaplan–Meier plots of the cumulative incidence of moderate-to-severe postoperative hypoxemia, which was defined as a PaO_2_/FiO_2_ ratio lower than 200 mmHg. **Figure S4.** Postoperative dyspnea, as assessed by the visual analog scale, in the two study groups. No inter-group differences were detected (ANOVA *p* = 0.97). A part of these results is displayed in Table 1. (PPTX 200 kb)

Additional file 2: Results of the univariate analysis on the factors associated with the development of postoperative hypercapnia. (DOCX 13 kb)

## Abbreviations

BMI: Body mass index
COPD: Chronic obstructive pulmonary disease
HFNC: High-flow nasal cannula
PEEP: Positive end-expiratory pressure
