# Supplementary material for: Early nasal high-flow versus Venturi mask oxygen therapy after lung resection: a randomized trial
Source: Crit Care. 2019 Feb 28;23:68. doi: 10.1186/s13054-019-2361-5 (PMC6396480; doi:10.1186/s13054-019-2361-5)
Supplement: Supplementary file 1 — Figure S1. Visual analog scale for the assessment of patient’s dyspnea. Figure S2. Mean (standard deviation) FiO2 in the two study groups (p = 0.23 for the inter-group comparison). Figure S3. Kaplan–Meier plots of the cumulative incidence of moderate-to-severe postoperative hypoxemia, which was defined as a PaO2/FiO2 ratio lower than 200 mmHg. Figure S4. Postoperative dyspnea, as assessed by the visual analog scale, in the two study groups. No inter-group differences were detected (ANOVA p = 0.97). A part of these results is displayed in Table 1. (PPTX 200 kb) [file 13054_2019_2361_MOESM1_ESM.pptx]

## Slide 1
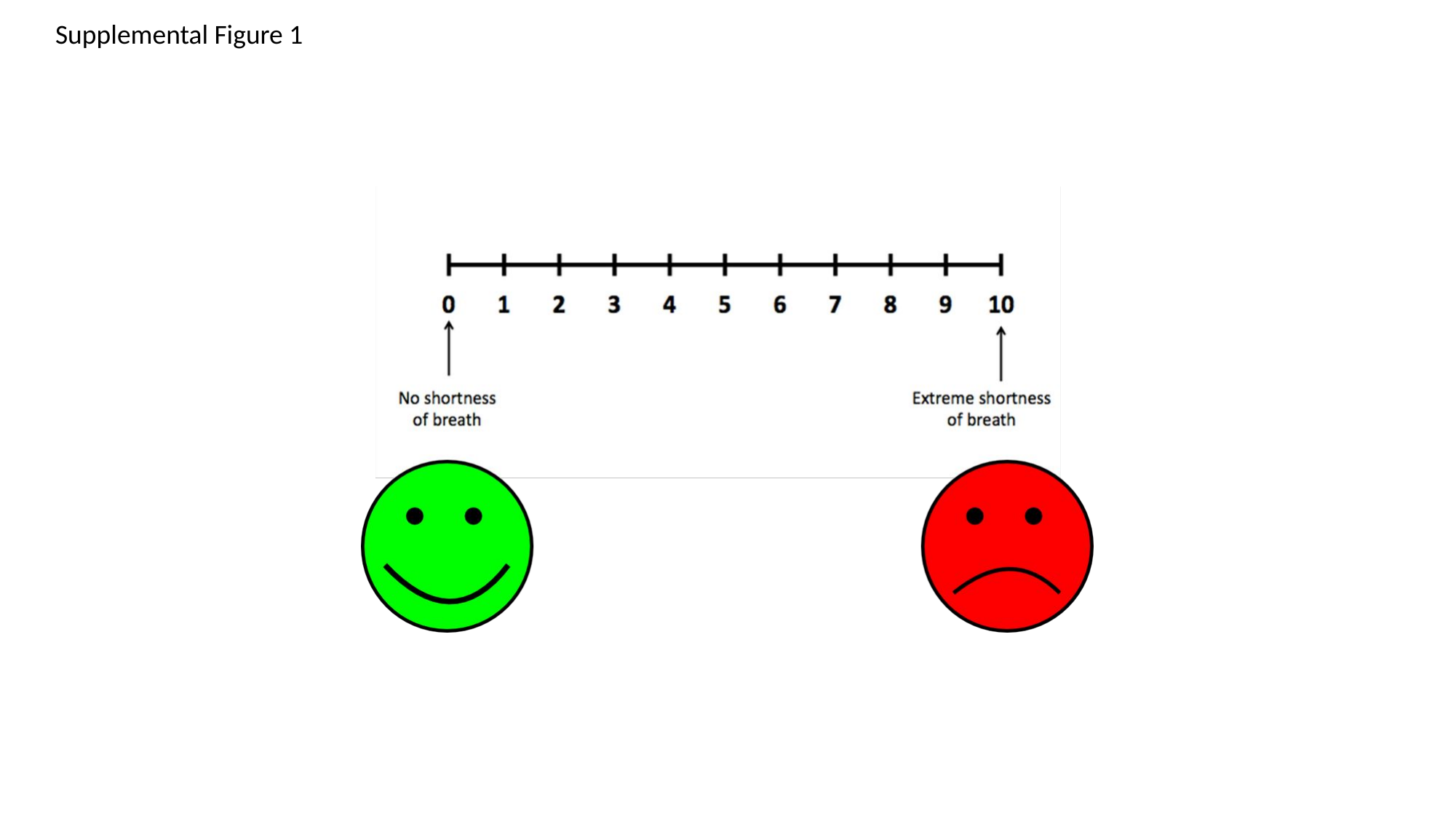

Supplemental Figure 1

## Slide 2
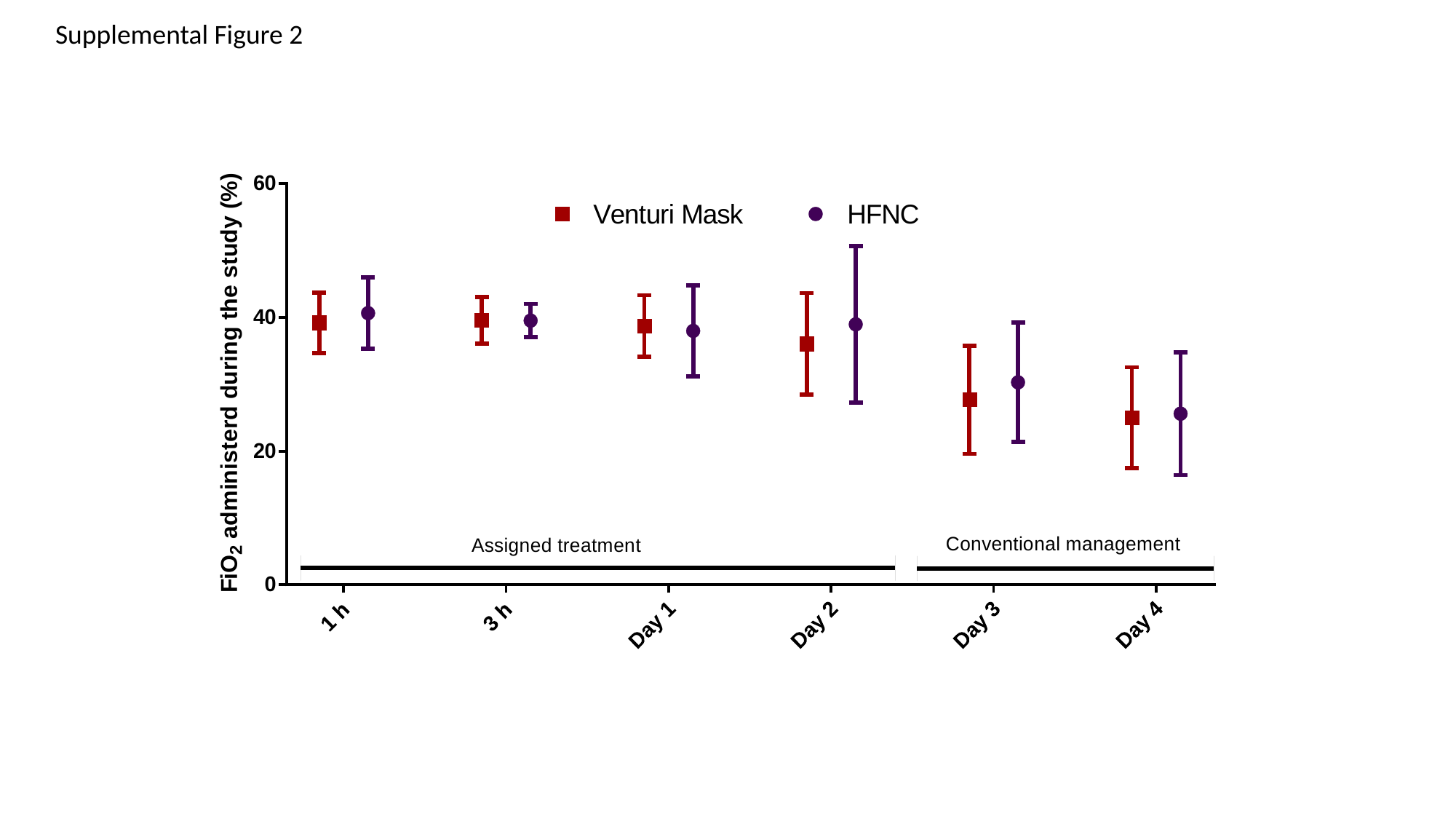

Supplemental Figure 2

## Slide 3
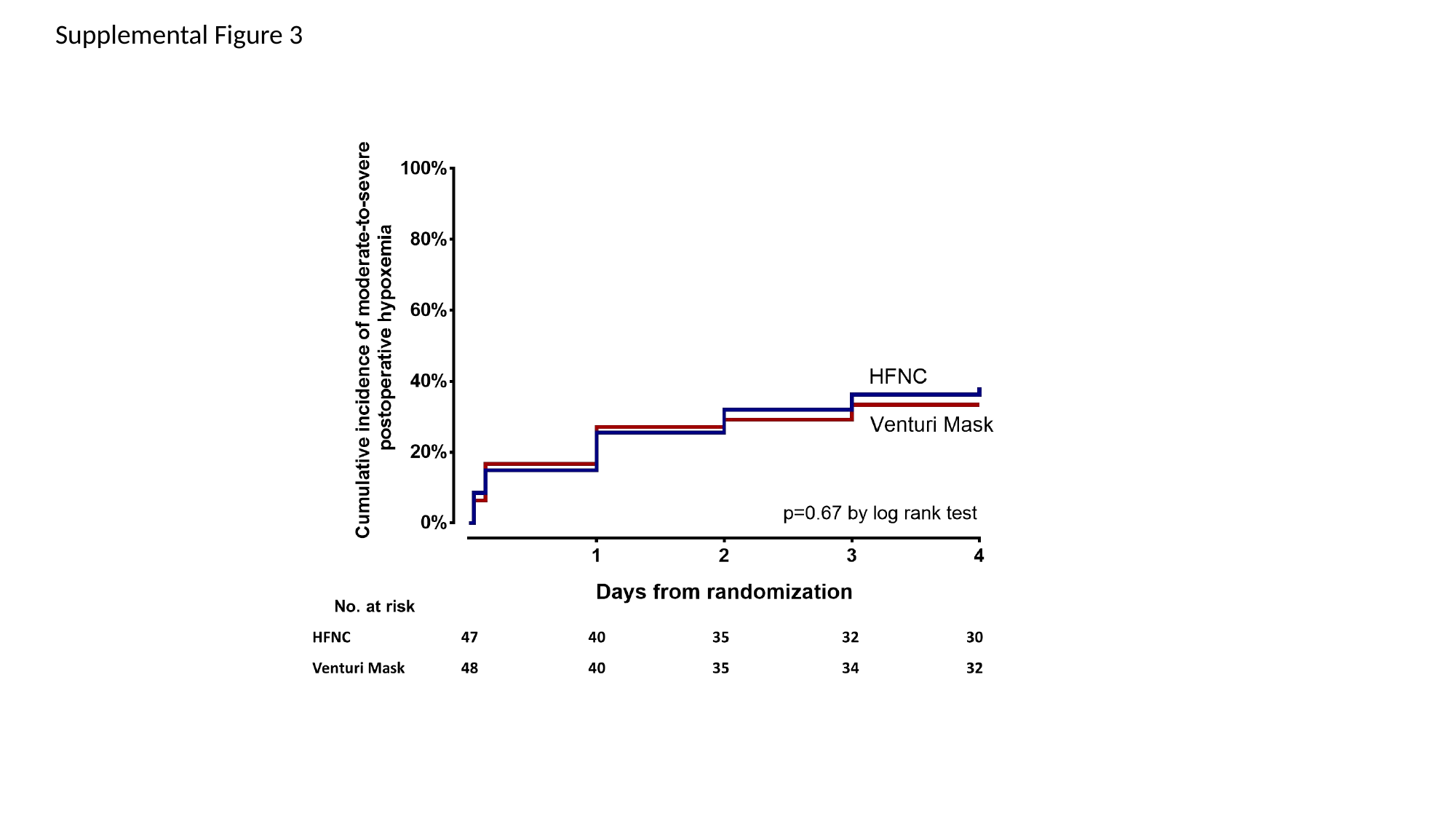

Supplemental Figure 3

## Slide 4
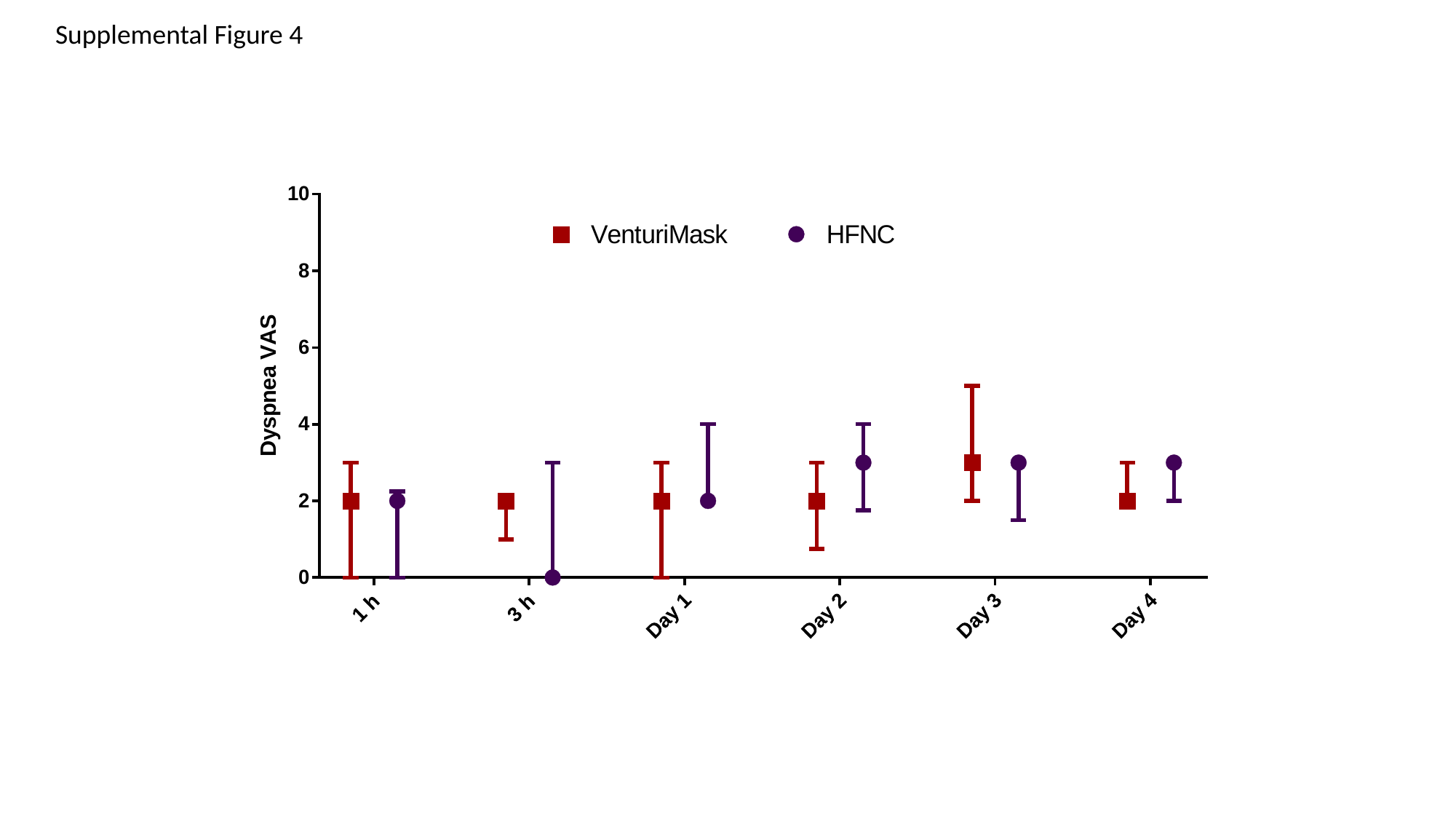

Supplemental Figure 4
