# Supplementary material for: Early nasal high-flow versus Venturi mask oxygen therapy after lung resection: a randomized trial
Source: Crit Care. 2019 Feb 28;23:68. doi: 10.1186/s13054-019-2361-5 (PMC6396480; doi:10.1186/s13054-019-2361-5)
Supplement: Supplementary file 2 — Results of the univariate analysis on the factors associated with the development of postoperative hypercapnia. (DOCX 13 kb) [file 13054_2019_2361_MOESM2_ESM.docx]

| **Supplementary table 1. Univariate analysis on the development of postoperative hypercapnia** | |
| --- | --- |
|  | p-value |
| Study treatment | **0.004** |
| Age | **0.11** |
| Induction therapy | 0.60 |
| Hypertension | 0.45 |
| History of cardiac failure | 0.31 |
| History of ischemic heart disease | 0.27 |
| Chronic obstructive pulmonary disease | 0.93 |
| Pulmonary infections in the month preceding surgery | **0.18** |
| Active smoking | 0.23 |
| Diabetes | 0.28 |
| Preoperative hemoglobin | 0.71 |
| Preoperative PaO_2_ | 0.45 |
| Preoperative PaCO_2_ | **<0.001** |
| Length of intraoperative mechanical ventilation | 0.36 |
| Length of surgery | 0.52 |
| Mean PaO_2_ during the 96-hour study period | 0.66 |
| Mean PaO_2_ while patients were receiving the allocated treatment | 0.60 |
